# Defining the Nature of Thermal Intermediate in 3 State Folding Proteins: Apoflavodoxin, a Study Case

**DOI:** 10.1371/journal.pcbi.1002647

**Published:** 2012-08-23

**Authors:** Rebeca García-Fandiño, Pau Bernadó, Sara Ayuso-Tejedor, Javier Sancho, Modesto Orozco

**Affiliations:** 1 Joint IRB BSC Program in Computational Biology, Institute for Research in Biomedicine, Barcelona, Spain; 2 Department of Organic Chemistry and Center for Research in Biological Chemistry and Molecular Materials, Santiago de Compostela University, Santiago de Compostela, Spain; 3 Centre de Biochimie Structurale, INSERM U1054, CNRS UMR 5048, Université Montpellier 1 and 2, Montpellier, France; 4 Departamento de Bioquímica y Biología Molecular y Celular, Facultad de Ciencias, Universidad de Zaragoza, Zaragoza, Spain; 5 Joint Unit BIFI-IQFR, CSIC, Spain, Biocomputation and Physics of Complex Systems Institute (BIFI), Universidad de Zaragoza, Zaragoza, Spain; 6 Departament de Bioquímica, Facultat de Biologia, Universitat de Barcelona, Barcelona, Spain; University of Illinois, United States of America

## Abstract

The early stages of the thermal unfolding of apoflavodoxin have been determined by using atomistic multi microsecond-scale molecular dynamics (MD) simulations complemented with a variety of experimental techniques. Results strongly suggest that the intermediate is reached very early in the thermal unfolding process and that it has the properties of an “activated” form of the native state, where thermal fluctuations in the loops break loop-loop contacts. The unrestrained loops gain then kinetic energy corrupting short secondary structure elements without corrupting the core of the protein. The MD-derived ensembles agree with experimental observables and draw a picture of the intermediate state inconsistent with a well-defined structure and characteristic of a typical partially disordered protein. Our results allow us to speculate that proteins with a well packed core connected by long loops might behave as partially disordered proteins under native conditions, or alternatively behave as three state folders. Small details in the sequence, easily tunable by evolution, can yield to one or the other type of proteins.

## Author Summary

A simplistic view of protein structure tends to emphasize the opposition between the native state and the denatured ensemble of unfolded conformations. In addition to these extreme conformations, proteins subjected to a variety of perturbations often populate alternative partly unfolded conformations, some of which are close in energy to the native state and, accordingly, can be populated under native or quasi-native conditions. There is increasing evidence that these “perturbed” conformations participate in protein function or, in some cases, are related to the outcome of folding diseases. We have used the “state of the art” molecular dynamics combined with a variety of experimental techniques to characterize for the first time, to our knowledge, the thermal intermediate of a three-state folding protein (apoflavodoxin). Based on our results we have been able to suggest a general mechanism of thermal unfolding in complex proteins and to determine interesting links between thermal intermediates and partially unfolded proteins.

## Introduction

In addition to the folded and unfolded states, many proteins may adopt stable conformations that display mixed properties of the native and denatured states. These conformations, usually known as intermediates, may appear under unusual external conditions (i.e. non-physiological pH, pressure or temperature), in the presence of high concentrations of certain cosolutes (denaturants, salts), or as a consequence of mutations [1]–[9], and are supposed to be populated during folding (and unfolding), especially in the case of medium or large proteins [10]. Such folding intermediates may be on-pathway, facilitating the reaction, or off-pathway, acting as traps that may lead to missfolding and even aggregation [11]–[12].

The occurrence of equilibrium intermediates is often associated with stress phenomena and can trigger pathological effects, such as spongiform encephalopathy and other types of amyloidosis [13]–[14]. This explains the existence of many physiological mechanisms designed to reduce their harmful effects, mainly by reducing the life time of these potentially dangerous conformations [15]–[17]. For some proteins, however, physiological roles have been postulated for their intermediate conformations and such possibility might be more common than originally believed [8], [18]–[21]. All these reasons explain the interest in understanding the nature of intermediates and the atomistic details that favours the transition from native to intermediate structures. Unfortunately, the study of intermediate conformations is much more difficult than that of native forms. Equilibrium intermediates can be detected *in vitro* as a deviation from two-state behaviour, i.e., non-coincident protein unfolding curves obtained with different experimental techniques [22], although their structures and energetic properties are more difficult to probe. Folding intermediates are elusive to X-ray crystallography and their normally small population and the extensive signal broadening compared to the native state difficult their analysis by means of NMR techniques [23]–[28]. As a consequence, structural information on intermediates is often obtained by using low-resolution techniques, often based on Φ-analysis [29], [30]–[31], or low-resolution spectroscopic or scattering data, which can give clues on the general shape of the protein, but not atomistic information. This explains the need and frequent use of simulation techniques, particularly molecular dynamics (MD) to try to gain atomistic details that are unreachable to experimental techniques [32]–[44].

Flavodoxins are a family of proteins essential for the survival of many human pathogens that has become one of the most studied models for protein folding and unfolding. They are mono-domain α/β proteins, with a parallel five-stranded β-sheet surrounded by five α-helices, and they carry a non-covalently bound FMN group which can be reversibly removed [45]. Several experimental studies on apoflavodoxins from the *Anabaena* [46]–[47], *Azotobacter* and several *Desulfovibrio* [48]–[50] strains have demonstrated the thermal unfolding of this protein follows a three-state mechanism, where a partly unfolded intermediate accumulates at moderately high temperatures. Using a variety of techniques applied to wild type and mutant proteins Sancho's group arrived to a low resolution picture of the thermal intermediate of the apoflavodoxin from *Anabaena* PCC 7119 [51]–[52] finding evidences that the intermediate is in fact close to the native structure, with the two hydrophobic cores well preserved, and with distortions probably located mostly in the loops and in one of the β-strands [53]. The overall dimensions of this thermal intermediate were characterized by small-angle X-ray scattering analysis, which suggests that the intermediate is slightly more extended than the native form, but clearly far from the expected situation of a random coil [54].

In this paper, we present a massive molecular dynamics (MD) effort for the study of the early stages of thermal unfolding of apoflavodoxin from *Anabaena* and for the characterization of its thermal intermediate. The study is especially challenging, since the slow folding dynamics of this protein (average transition times in the order of 10^1^–10^2^ millisecond makes impossible the use of pure force-approaches based on atomistic potentials, which would require second-scale trajectories. Furthermore, many attempts to use coarse-grained potentials failed to sample structures reproducing experimentally known intermediate properties and unfolding pathways, while equilibrium dynamics obtained from coarse-grained potential seems stiffer, but qualitatively similar to that expected from MD simulations (data not shown but available upon request). Accordingly, we decided to use a hybrid approach, based on the use of microsecond scale atomistic MD, supplemented by low- resolution spectroscopic and scattering data and previously derived Φ-analysis. The approach allowed us to characterize with atomistic detail the ensemble of conformations that define the intermediate as experimentally detected in melting experiments. With this synergistic approach the mechanism that drives the transition from native to intermediate and most likely the early stages of the thermal unfolding of the protein were explored.

## Methods

### Molecular dynamics simulations

The crystal structure of *Anabaena* apoflavodoxin deposited in the Protein Data Bank with reference 1FTG [51] was used as starting conformation for our simulations. Crystallographic SO_4_^2−^ was conserved and simulated together with the protein [in the X-ray structure of Anabaena apoflavodoxin (crystallized in high ammonium sulphate concentration), a sulphate ion is bound, mimicking the FMN phosphate, which opens the possibility that the native conformation in this region is a consequence of the binding of the ion], the rest of ions 24 Na^+^ and 6 Cl^−^ which are needed to neutralize large values of electrostatic potential around the protein were added using CMIP calculations implementing Poisson-Boltzman potentials [55]. The resulting systems were then solvated by around 7600 TIP3P water molecules [56], partially optimized, thermalized to 300 K (Nose-Hoover thermostat) and equilibrated using our standard protocol [57], followed by additional 50 ns of post-equilibration. Ten randomly selected snapshots (separated by at least 1 ns) were selected from the last 20 ns of the equilibration trajectory to generate the starting coordinates of ten replicas of the protein in water at T = 300 K. To increase diversity in the ensemble of the native form velocities were randomized and each replica was re-equilibrated for 5 ns prior to 0.2 µs isothermic-isobaric production simulations (T = 300 K, P = 1 atm). The structure obtained at the end of the 50 ns equilibration at T = 300 K was heated slowly (0.5 ns) to 368 K and equilibrated at this temperature for additional 10 ns, followed by 2 µs simulation using isothermic-isobaric conditions (T = 368 K, P = 1 atm). Periodic boundary conditions and Particle Mesh Ewald calculations were used to deal with long-range effects [58]. RESPA (Multiple time step) [59] with a minimum time step of 1 fs was used in conjunction with RATTLE [60] algorithms for maintaining bonds involving hydrogen atoms at equilibrium distances.

Multi-microsecond trajectory at high temperature suggests that under the simulation conditions the unfolding trajectory reaches conformations which reproduce known properties of the thermal intermediate in less than 200 ns (see Results). Thus, to enrich our trajectories with the intermediate sate we performed 50 independent simulations starting from 50 different snapshots of the solvated protein extracted every nanosecond during the first 50 ns of the long T = 368 K simulation. Velocities in each snapshot were randomized and after 5 ns re-equilibration the 50 independent trajectories were followed for 0.2 µs using identical simulation conditions, representing an aggregate time in the replicas of 12 µs. Such meta-trajectory was analyzed to determine the nature of the intermediate by confronting collected structures with experimental observables of the intermediate state.

All MD simulations were carried out with NAMD 2.6 [61]–[62] computer program using the CHARMM27 [63]–[64] force field using the *MareNostrum* supercomputer at the Barcelona Supercomputer Center.

### Trajectory analysis

Snapshots were saved every picosecond and submitted to a large variety of analyses. Basic geometrical descriptors were determined using the *ptraj* module of AMBER9 [65]–[67], clustering was done in function of the RMSd of the clustered structures using the MMTSB Tool set [68] and representative structures of the clusters were determined as those closer to the centroid of each cluster. Secondary structure assignment and solvent accessibility of the representative structures of each cluster were calculated independently using the program PROCHECK [69]. Theoretical changes in the UV spectrum of the protein related to unfolding were determined by analysing the solvent accessible surface of the four Trp (SAS_Trp_) and using four references: i) the crystal structure, ii) the ensemble obtained in MD simulations at room temperature, iii) four isolated Trp and iv) the protein after 50 ns of MD simulation at T = 500 K (where it reaches RMSd>15 Å from X-ray structure and all structural signatures are lost). SAS were computed using the NACCESS [70] program with standard values for protein and solvent particles.

Essential dynamics (ED) [71] was done to determine the nature of the easiest deformation movements in the native and intermediate states of the protein and to determine the overlap between the essential deformation modes of the protein and the native<$>\raster="rg1"<$>intermediate transition vectors. For this purpose covariance matrices were calculated for the native and intermediate ensembles (using a common reference system defined by the structurally conserved regions of the protein). Such covariance matrix was diagonalized to obtain a set of eigenvectors (the essential deformation modes) and the associated eigenvalues (the amount of variance associated to each eigenvector). The similarity between the essential space of native and intermediate was compared using Hess metrics [72]–[73] taking 50 eigenvectors as a common essential space (at least 90% of variance explained in each ensemble):
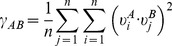
(1)where n is the dimension of the essential space, A and B are two ensembles and 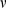 stands for the eigenvectors. Considering the relative size of the protein and the essential space, any 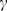
>0.1 signals a statistically significant similarity [74].

The relative similarity between two essential deformation spaces was computed using [73]:
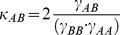
(2)where the self-similarity indexes 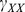 where obtained by comparing two different parts of the ensemble. Relative similarity index corrects absolute metrics by the intrinsic noise of MD simulations. A 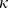 value close or even greater than 1 indicates that considering the noise of the trajectories the two ensembles are identical.

The transition from the intermediate to the native states was obtained by taking the first eigenvector calculated by principal component analysis of a meta-ensemble obtained by mixing an equal number of snapshots of the intermediate and the native state. The overlap between the intermediate essential dynamics and the intermediate→native transition vector was determined as:
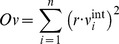
(3)where *Ov* is the overlap (maximum equal to one), *r* is the transition vector and *int* stands here for the intermediate ensemble.

### φ-values

Experimental φ-values profiles were taken from a previous work by Sancho's group [75], [52], [53]. Theoretical estimates were derived by individual φ_i_^calc^ values (i stands for a residue) determined as the fraction of native contacts, N_i_, made by that residue in the MD with respect to those found in the crystal structure, N_i_^nat^ i.e., φ_i_^calc^ = N_i_/N_i_^nat^ [76]. Comparison between experimental and simulated φ values was extended to all residues with φ_i_<1 except for residues in helix 3, where experimental uncertainties in the determinations were large [75]. The ability of a structural ensemble to satisfy the experimental Φ-value profile was studied by analyzing the sum (over all residues) of the difference between predicted and simulated Φ-values:
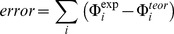
(4)


### Small-angle X-ray scattering measurements and analysis

SAXS experiments were performed on the high brilliance beamline ID02 at the European Synchrotron Radiation Facility (ESRF, Grenoble, France). An apoflavodoxin sample at 1 mg/ml concentration was prepared in 50 mM Mops buffer at pH 7. Several SAXS curves were acquired with a momentum transfer range of 0.07<*s*<0.31 Å^−1^ at a broad range of temperatures (6–67°C). Solutions were pushed in a capillary into the chamber where they were equilibrated for five minutes. An equivalent protocol was applied to measure buffer profiles. Ten successive frames of 1 s each were acquired for both sample and buffer. Each frame was inspected and the presence of protein damage was discarded. The different scans at each temperature were averaged and subtracted from their buffer counterpart using standard protocols with PRIMUS [77]. The forward scattering, I(0), and the effective radius of gyration, R_g_, was obtained from the scattering profiles using the Guinier's approximation [78] assuming that, at very small angles (*s*<1.3/R_g_), the intensity can be represented as I(*s*) = I(0) exp(−(*s*R_g_)^2^/3).

SAXS curve measured at 26°C was used to evaluate MD trajectories in native conditions. The evaluation of trajectories in denaturing conditions was performed with the curve obtained from the Multivariate Curve Resolution by Alternating Least Squares (MCR-ALS) analysis of the SAXS dataset measured at the complete range of temperatures used to follow thermal denaturation of apoflavodoxin [54]. Principal Component Analysis (PCA) of the temperature variation SAXS dataset identified three components in the apoflavodoxin denaturation process that were assigned to the native the unfolded, and an intermediate states. MCR aims at finding the pure SAXS curves of these coexisting species in solution as well as the evolution of the relative concentration of these species upon environmental changes. The decomposition is obtained by solving the matrix equation
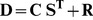
(5)where **D** is the SAXS data matrix, **C** is the matrix describing the contributions of the *N* components, **S^T^** is the matrix describing the instrumental responses of these *N* components, and **R** accounts for the residuals of the fitting. Details of MCR-ALS approach and its application to SAXS data can be found in the original publications. [79]–[82]. Due to the post-processing nature of the SAXS profile of the intermediate, no experimental errors are associated to the derived intensities. A homogeneous 7% of error was assumed for each of the intensities of the curve. The agreement of SAXS profiles with three-dimensional structures of the MD trajectories was evaluated with CRYSOL [83] using default parameters. The χ-value of the fitting between experimental and theoretical curves is used as a measure of the quality of fitting (the smaller the χ-value, the better the agreement). Note that due to the de-convolution process and the use of a small homogeneous error in the intermediate, larger χ-values are expected in the fitting of the intermediate than to that of the native state.

### UV spectra

Near-UV absorbance spectra of apoflavodoxin [51] at different temperatures were recorded from 250 to 310 nm in a *Chirascan* spectropolarimeter (from *Applied-Photophysics*) using 30 µM protein solutions in 50 mM Mops, pH 7 in a 4 mm path-length cuvette. The absorbance spectra of native, intermediate and unfolded *Anabaena* apoflavodoxin were then determined by deconvolution of spectra recorded at different temperatures, using equation:
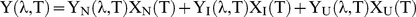
(6)where the observed absorbance value at a given wavelength and temperature, Y(λ,T), is a linear combination of the values of the different states, Y_i_(λ,T) and of their populations, X_i_(T) [84]. On the other hand, the populations are calculated at each temperature from the free energy values Δ*G*_1_ and Δ*G*_2_ previously obtained by global fitting to the sequential three-state model of unfolding curves recorded using absorbance, fluorescence and circular dichroism [53].

## Results

### Equilibrium trajectories at room temperature

**Figure 1:**
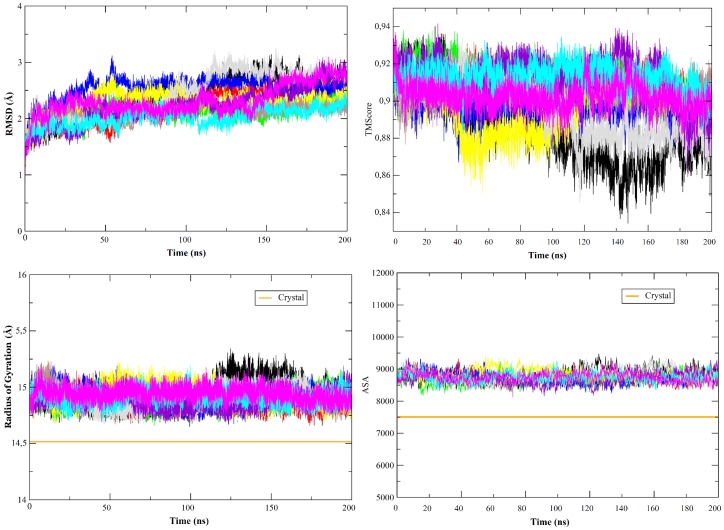
Evolution of different metrics along the 10 trajectories of apoflavodoxin at room temperature. TOP: All atoms root mean square deviation (LEFT) and TM-score^44a^ (RIGHT) from crystal structure (both in Å). BOTTOM: Radii of gyration (LEFT, in Å) and solvent accessible surface (RIGHT, in Å^2^). Orange lines represent the values found in the crystal structure (PDB code 1FTG).

Ten independent 200 ns long MD simulations suggest that the equilibrium structure of the protein in solution is close to that found in the crystal, without any clear unfolding tendency (Figure 1). The RMSd of trajectory from the crystal structure are always below 3 Å for all replicas, and seems quite stable after the first 10–40 ns where protein relax from lattice contacts existing in the crystal structure (see Figure 1). The general shape of the protein and the structural core is fully maintained (see Tm-score plot in Figure 1) and the most significant deviation from crystal state is a small expansion of the protein as a result of the removal of lattice constraints, a behaviour very commonly found in massive MD simulations of the proteome [85]–[86], which is visible in a small (in average around 0.5 Å see Figure 1) increase in radii of gyration (which happens already in the post-equilibration phase) as well as in an increase around 13% in the solvent accessible surface without changes in the polar/apolar SAS (see Figure 1 and Suppl. Figure S1). This slight increase in the size of the protein when liberated from lattice constraints is reflected in a small increase in the Trp accessibility, a parameter that correlates with the UV spectra [87] of the protein (see Suppl. Figure S2). However, all changes in size and shape of the protein upon transferring from crystal to solution are small. Not surprisingly then, the scattering properties computed from the 2 µs ensembles agree very well with the experimental SAXS curve, and also math the conformational preferences indicated by the X-ray structure (see Suppl. Figure S3).

**Figure 2:**
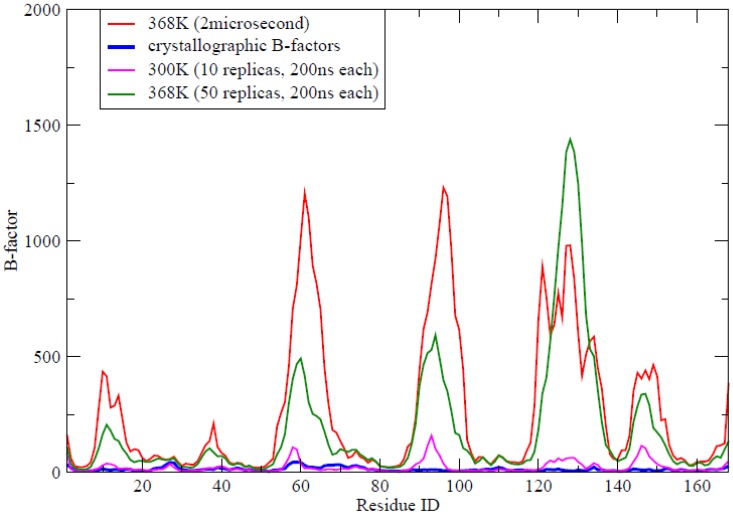
B-factors profile (Å) for apoflavodoxin obtained from simulations: i) the control meta-trajectory at 300 K, ii) the large unfolding trajectory at 368 K, iii) the meta-trajectory at 368 K and iv) the profile reported in the crystal structure (PDB code 1FTG).

Contacts between residues are massively preserved (see Suppl. Figure S1) and the few native contacts which are transiently lost are typically replaced by alternative contacts with neighbouring residues (see Suppl. Figure S1). Both α helices and β sheet elements remain fixed at crystal values, while there is a conversion of a portion of residues in β turn into coil conformations (see Suppl. Figure S1), which is localized in the loop regions. In fact, analysis of B-factors (Figure 2) obtained in the 2 µs meta-trajectory reveals that the regions of larger flexibility are located around the loops of the protein. It is worth noting that most of these loops appear with large B-factors in the crystal structure. However, two loops which are flexible in the simulation (loop 90–100, contributing to binding the cofactor; and loop 120–135, characteristic of long-chain flavodoxins and involved in the binding of partner proteins) appear with low B-factors in the crystal. Analysis of different crystal structures of this protein in PDB (including 1FTG used here as starting conformation) reveals that in the crystal all these loops are directly or indirectly constrained by intermolecular packing contacts, which suggests that the largest mobility found in our simulations cannot be considered a simulation artefact (see Suppl. Figure S4).

**Figure 3:**
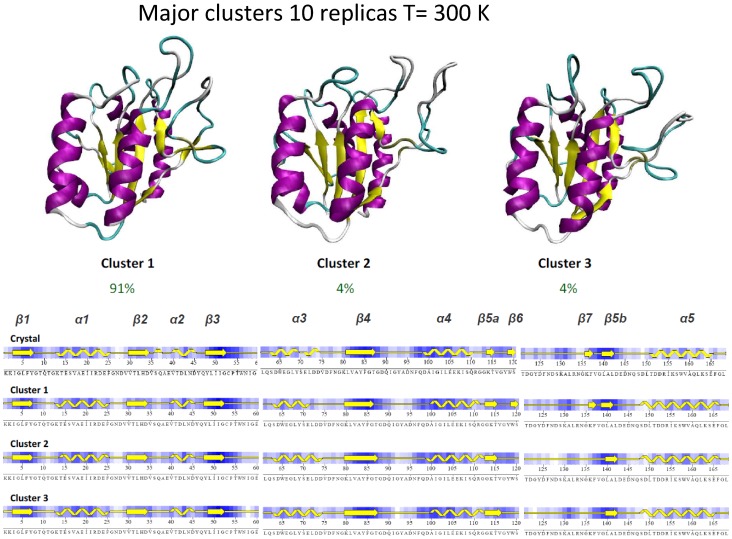
Major clusters obtained (cluster radii 2.5 Å; see Methods) from the meta-trajectory of apoflavodoxin at room temperature. The typical secondary structure content of each of cluster is shown, with the X-ray structure as reference.

Cartesian cluster analysis (Figure 3) reveals that around 91% of the time trajectories are sampling the same conformational basin, which is very close to the crystal structure (RMSd to the crystal 1.5–2.5Å). The trajectory also populates two alternative basins (two clusters with population 4% each; RMSds to crystal 2.0–2.5Å) that only differ in the conformation of the long loop characteristic of the long-chain flavodoxin family (including β_6_ and β_7_; positions 120–135) and, at a minor extent, in the 90–100 loop (that connecting β_4_ and α_4_). Conformational changes in the loops yield to a marginal loss of native contacts in the region (see Figures 2 and 3), without further changes in the global structure. In summary, extended MD simulations demonstrate that selected force-field and simulation conditions are able to represent the folded form of the protein, which seems to be quite rigid except for local movements in the aforementioned loops.

### Extended unfolding at high temperature

It is never clear what is the effective temperature in a classical MD simulation, since it is force-field dependent [88]–[89]. It is then almost impossible to define a simulation temperature as to guarantee that a finite time simulation will populate the experimentally characterized thermal intermediate. Thus, as described in Methods, we decided to locate (by comparison with experimental data) the intermediate as a transient conformational ensemble populated during unfolding at high temperature (below water boiling point).

**Figure 4:**
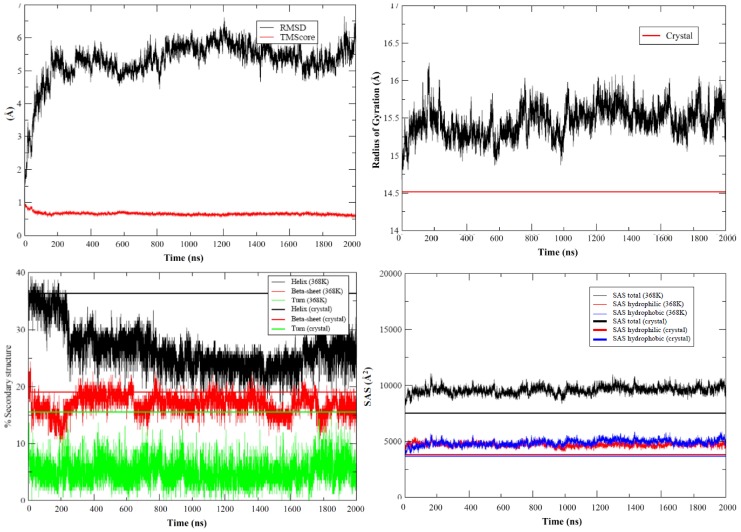
Evolution of different metrics along the 2 µsec unfolding simulation of apoflavodoxin at 368 K. TOP/LEFT: All atoms root mean square deviation and TM-score from crystal structure (both in Å). TOP/RIGHT: Radii of gyration (in Å). BOTTOM/LEFT: content of secondary structure. BOTTOM/RIGHT Solvent accessible surface (total, hydrophobic and hydrophilic, all in Å^2^). Reference lines represent always the values found in the crystal structure (PDB code 1FTG).

**Figure 5:**
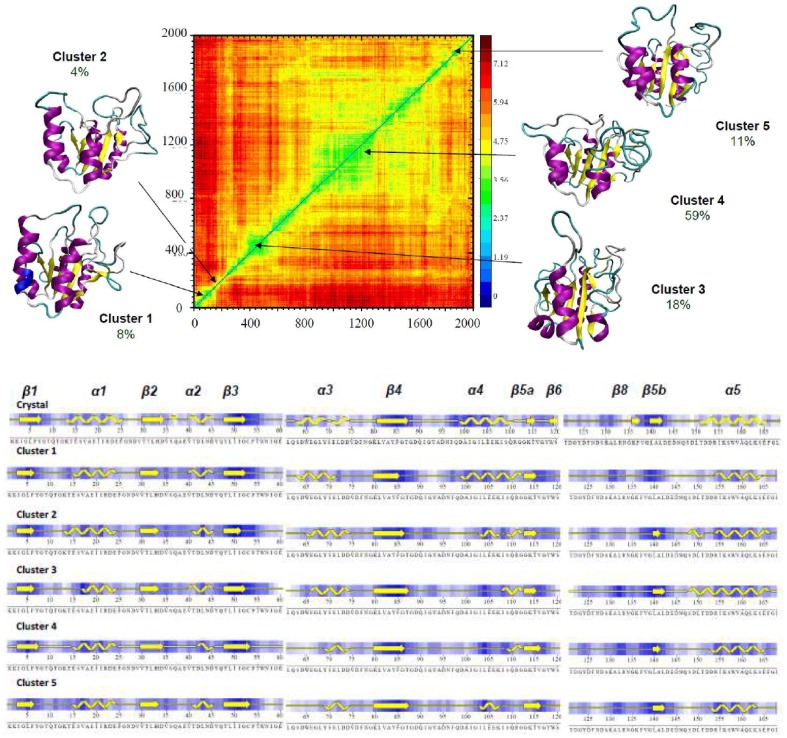
Cross-root mean square deviation plot for the 2 µsec unfolding simulation of apoflavodoxin at 368 K (RMSd color code is in Å). The central structure of the different clusters (cluster radii 4.6 Å) sampled during the trajectories are projected into the cross RMSd plot. The typical secondary structure content of the structures in the different clusters is shown with a reference to X-Ray structure (PDB code 1FTG).

The increase in the temperature does not lead to complete protein unfolding in 2 µs (see Figures 4–5), something that could be expected only in very fast-folder proteins, typically small proteins with simple kinetic folding mechanisms. The maintenance of TM-score and the hydrophobic solvent accessible surface demonstrate that the protein core is preserved even until 2 µs of trajectory at high temperature. However, although the general fold is maintained, structural distortions from native structure are significant at the end of the simulation (as noted in the large RMSd) and affect key elements of α and β secondary structure (Figures 4–5). Major distortions are first located at the loops, as expected from native simulations (see above), but are later propagated to the neighbouring elements of secondary structure (see Figures 4–5). Thus, the large movements of loop 90–100 lead to distortions in neighbouring helix α_4_, which is shortened in 0.2–0.5 µs part of the trajectory and is almost completely lost at the end of it. Similarly, distortions in the long loop 120–135 produce early in the unfolding trajectory the disruption of small β-sheet elements β_6_, β_7_ and β_5b_ and the shortening of terminal helix α_5_. Large movements of other smaller loops like 53–62 and 75–80 lead also to distortions of neighbouring secondary elements (for example helix α_3_), but this happens late in the trajectory and is less dramatic than those noted above. Clearly, our long simulation has not statistical power to describe the intermediate, but suggests a general picture where the perturbation in the loops corrupts in a first step short elements of secondary structure, which has no impact in global structure, but later the α-helices segments are compromised which should eventually yield to the complete unfolding of the protein in longer time scales.

Cartesian cluster analysis reveals significant population (more than 100 ns) of 5 structural families along the 2 µs trajectories (Figure 5), which illustrates the increasing level of deformation gained along the simulation. It is tempting to assign the most populated family (cluster 4) as the putative intermediate, but as discussed above there is no guarantee that effective microscopic simulation temperature matches the experimental macroscopic temperature at which the intermediate is detected. Accordingly, we cannot be sure which family represents better the intermediate ensemble and we do not know at which time frame intermediate is populated during our MD unfolding simulations. Clearly, comparison with experimental observables can help to locate the intermediate in our ensemble.

**Figure 6:**
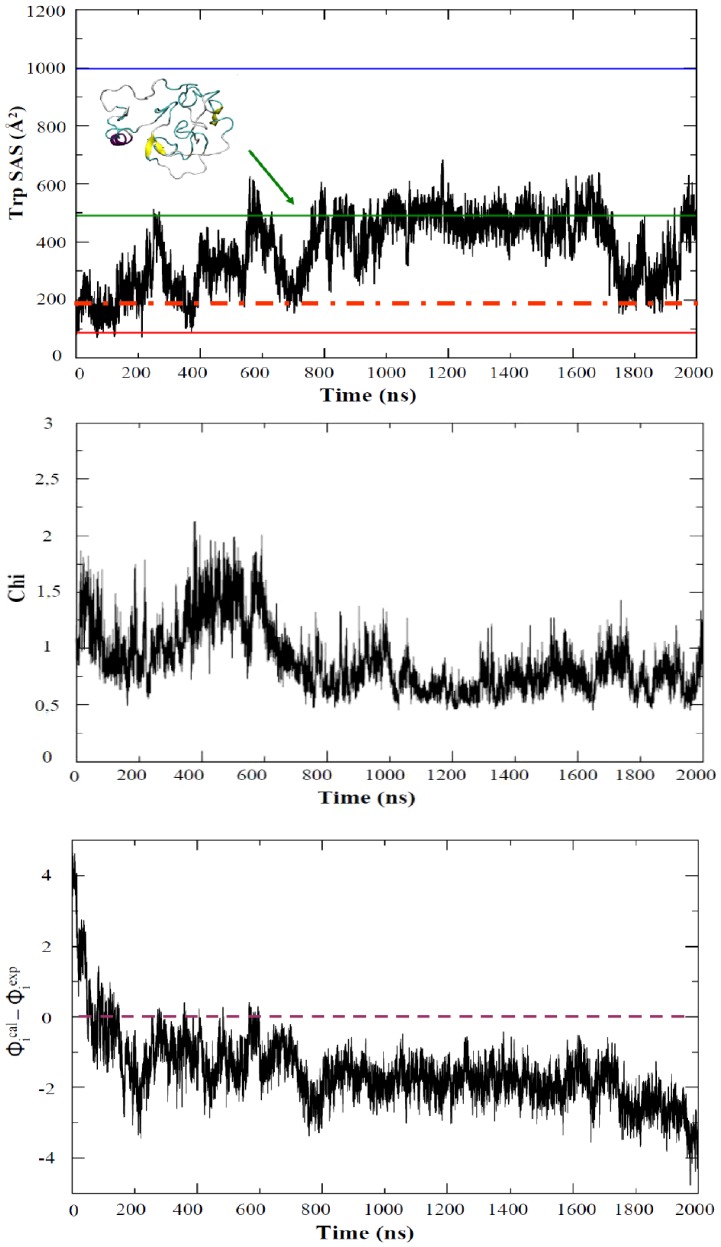
Evolution of different “experimental observables” that can be derived from the 2 µsec unfolding simulation of apoflavodoxin at 368 K. TOP: Solvent accessible surface of the 4 Trp of the protein; reference lines correspond to crystal structure (solid red), low temperature MD meta-trajectory (dashed red), a highly unfolded structure obtained by extreme heating of the protein (in green) and four fully exposed Trp (blue). MIDDLE: Evolution of the merit function for fitting experimental and MD-simulated small angle scattering profile (see text). BOTTOM: total difference between experimental and simulated Φ values, where positive values indicate structure too close to the native state and negative values signals too unfolded conformations.

The UV spectra determined experimentally for the intermediate (see Methods) is very similar to that of the native state, without the blue shift in the spectra which is clear in the unfolded state (see Suppl. Figure S5). Thus, we can be quite sure that the exposure of Trp side chains has not changed much from native to intermediate state. Based on this criteria the intermediate is detected during the beginning of the simulation (around 0.2 µs; Figure 6), while structures sampled at the second half of the trajectory yield too exposed Trp to justify experimental spectra. The SAXS spectra of the intermediate is well reproduced in the region 0.1–0.3 µs and later in the second half of the trajectory (as noted in χ values in Figure 6). Finally, the Φ–profile (see Methods) computed experimentally is well reproduced in the 0.1–0.2 µs region, while structures collected before are too “native-like” and those collected later have advanced too much in the unfolding pathway. In summary, comparison with experimental data strongly suggests that the intermediate is going to be closer to clusters 1–3 than to the most populated cluster 4 (see Figure 6), and that it is reached quite fast (around 0.2 µs) during our unfolding simulation.

### Ensemble simulations at high temperature

**Figure 7:**
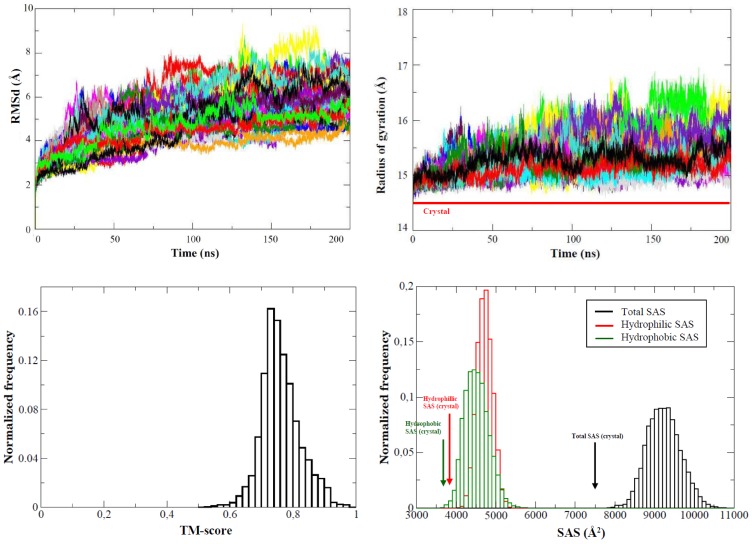
Evolution of different metrics along the 50 0.2 µsec trajectories of apoflavodoxin at T = 368 K. TOP/LEFT: All atoms root mean square deviation from crystal structure (in Å). TOP/RIGHT: Radii of gyration (in Å). BOTTOM/LEFT TM-score (from the crystal) distribution (in Å). BOTTOM/RIGHT: histogram of solvent accessible surface (total, hydrophobic and hydrophilic all in Å^2^). When reference lines appear they correspond to crystal structure.

**Figure 8:**
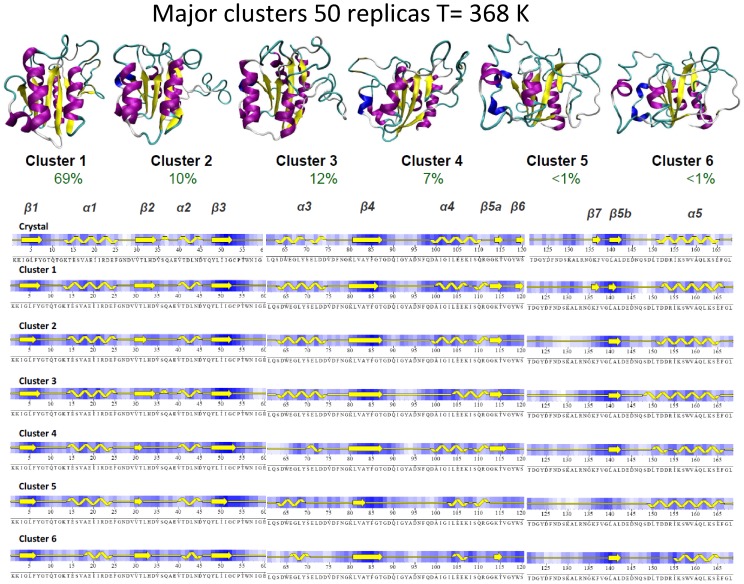
Major clusters (radii 5.5 Å) obtained in the meta-trajectory at 368 K. The percentage of population of these clusters and their typical secondary structure content are displayed.

Following the findings obtained from the analysis of the 2 µs trajectory, which suggested that native→intermediate transition happens early in the simulation, we performed 50 independent 0.2 µs trajectories, which combined provide us a 10 µs ensemble enriched in the intermediate state. All the different trajectories advance towards protein denaturation (see Figure 7), with a range of velocities that show a normal distribution with unfolding velocities ranging from 0.4 to 0.8 nm RMSd/0.2 µs. The lack of unusually slow or fast unfolding pathways [90] suggests the existence of a unique mechanism for the transition from folded to intermediate state under the selected simulation conditions, which is characterized by first a focalization of structural deformations in loops (Figure 7) and later a transfer of such perturbation to the surrounding elements of secondary structure (see Figure 8), matching the general unfolding trends found in the 2 µs trajectory.

Cartesian clustering of the 10 µs meta-trajectory allowed us to detect six major “states (clusters)”, four of them with populations above 5%. Not surprisingly, the most populated one (69% of meta-trajectory) is that describing a near-native conformation, which appears populated in the beginning of all the individual trajectories. As the unfolding progresses, partially unfolded conformations, characterized by distorted loop conformations and partial losses of neighbouring secondary structure become populated (Figure 8). Thus, in structures assigned to cluster 2 (10% meta-trajectory, populated in 55% trajectories) the large movements of the long loop (120–135) have led to the loss of short β strand elements β_6_ and β_7_. Ensembles represented by clusters 3 and 4 (12% and 7% meta-trajectory, populated in 65% and 45% individual trajectories) are characterized by an advance in the distortion produced by loop oscillations, either to the helix α_4_ (cluster 3) or the helix α_3_ (cluster 4). Finally, the minor clusters 5 and 6 represent much more distorted conformations, where a significant amount of secondary structure is lost and the departure from native basin is quite evident (Figure 8). Clusters 5 and 6 account for less than 1% of the entire meta-trajectory and are sampled only in two of the individual trajectories (one for each), which suggest that they do not fit the experimental requirements of the intermediate.

**Figure 9:**
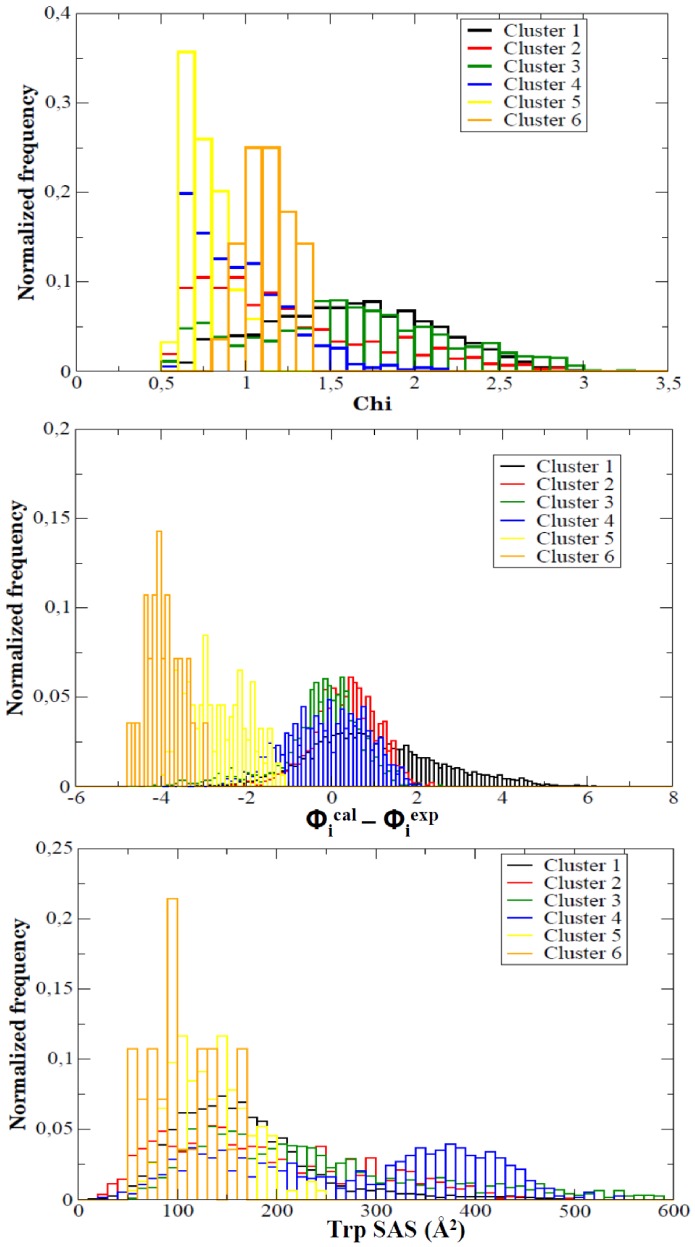
Histogram representation of the distributions of “experimental observables” obtained from the different clusters. From TOP to BOTTOM: fitting to SAXs curve, Φ-value error and Trp solvent accessible surface (see text and Figure 6 for details).

It is very tempting to try to identify one of the above mentioned clusters with the thermal intermediate, but analysis of the individual trajectories show that in reality clusters 2–4 and part of structures assigned to cluster 1 interchange in a fast way and share many common characteristics, with a well conserved central core and largely distorted loop regions. The fast and large movement of such loops (and neighbouring secondary elements) generates a large dispersion in the structures when projected into the Cartesian space, which is reflected in the different assignment of structures to different clusters, when they share many key structural characteristics. It is also worth to note that structures which are within the same cluster can yield very different values of some experimental observables (see Figure 9), while structures very distant in terms of RMSd, and accordingly assigned to the different clusters can be indistinguishable in terms of experimental observables (see Figure 9). In summary, it seems that the intermediate cannot be represented as a small ensemble defined as a narrow basin centered in a well-defined structure, but as a wide ensemble of conformations that cover a wide range of Cartesian space, but that share a common conformational core.

## Discussion

### The thermal intermediate

We interrogate our 10 µs ensemble to determine how many of these structures fulfill all the experimental requirements of the ensemble known experimentally for the thermal intermediate. Considering a loosely criteria (SAS_Trp_ between 100 and 300 Å^2^, fitting the SAXs curve with a χ below 1.5 and fitting the Φ-value profile with absolute accumulated error below 2) almost 30% of the ensemble is annotated as intermediate. If we assume that experimental measurements for the intermediate are very accurate and use a much more restrictive criterion (SAS_Trp_ between 100 and 300 Å^2^, χ<1.0 and Φ-error<1.0) the intermediate ensemble is reduced to around 10% of the meta-trajectory. Such an ensemble is contributed by all individual trajectories and is proportionally enriched with structures assigned to clusters 2–4, with no contribution of clusters 5 and 6.

**Figure 10:**
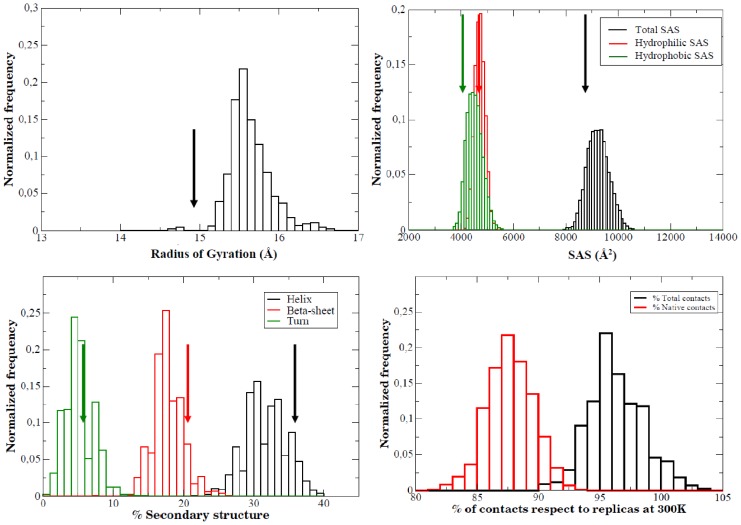
Histogram representation of different structural descriptors for the intermediate ensemble. TOP/LEFT: radii of gyration (in Å); TOP/RIGHT: solvent accessible surface (total, hydrophobic and hydrophilic, all in Å^2^); BOTTOM/LEFT: secondary structure content; BOTTOM/RIGHT: native and total inter-residue contacts. All reference arrows correspond to crystal values.

**Figure 11:**
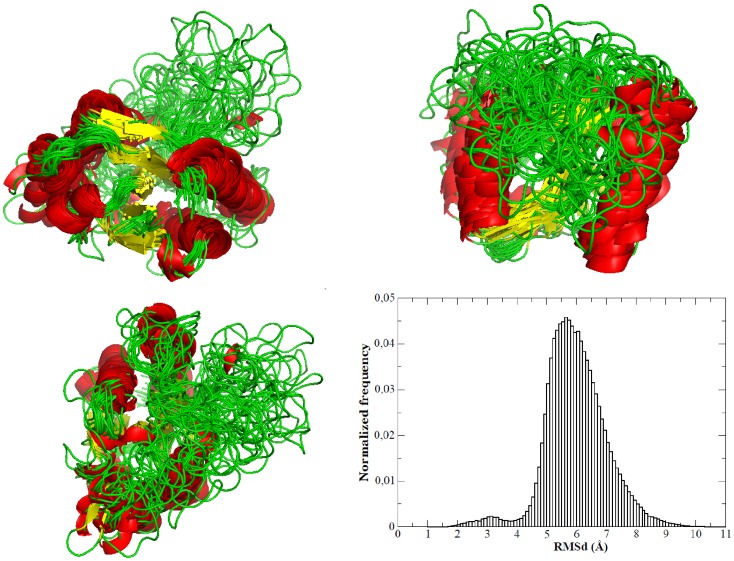
Three different views of the intermediate ensemble (10 randomly selected structures are superimposed) and the distribution of the root mean square distribution between different structures in the ensemble.

When analyzed, the intermediate sampling shows a quite interesting picture of the structure that is transiently populated during thermal unfolding of the protein (Figures 10–11). The structure has enlarged with respect to the solution ensemble and hydrophobic solvent accessible surface has increased significantly, a fingerprint of a partially unfolded structure. A significant number of native contacts (defined as those present in the solution ensemble) are lost, especially those involving the protein loops, which have disappeared completely (Suppl. Figure S6). However, the structure maintains still many native inter-residue contacts, mostly located in the central core, where the amount of secondary structure has decreased, but is still quite significant (Figures 10–11). Clearly, analysis of the results demonstrates that the intermediate is not an alternative structure of the protein, but has to be represented as a wide ensemble (average RMSd between structures in the ensemble is around 0.6 nm; Figure 11). Two broad regions can be easily recognized in the protein: the central core, where the native fold is well preserved and the loops (including the long loop hosting a small β-sheet encompassing strands 6 and 7), which adopt a canonical random coil confirmation (Figure 11). It is very interesting to realize that the large flexibility movements governing the essential dynamics in the intermediate ensemble are already a maximization of the intrinsic deformation pattern of the native state of the protein (absolute similarity (γ) = 0.52; relative similarity (κ) = 0.76, see eqs. 1 and 2), as it was already suggested by B-factor distributions (see Figure 2). Altogether, the intermediate fits perfectly in the definition of a partially disordered protein with a solid-like core and a liquid-like external loop core. It is very encouraging that such a representation of the intermediate fits well with the picture derived from the analysis of the NMR spectra of a mutant, which is believed to adopt intermediate-like conformation under native conditions [91].

Our MD simulations suggest a quite complete picture of the initial stages of the thermal unfolding of apoflavodoxin, which might be common to other proteins having long loops stabilized by weak contacts. Thus, under native conditions the protein has an intrinsic tendency to become a partially disordered protein, but several loop-loop contact keep the potentially flexible part of the protein reasonably organized. When the temperature increases these loops gain kinetic energy and in a quite short period of time become random coils (see Figure 2). The anchoring points of the loops, with the exception of short β-sheet elements, are very stable and held together the core of the structure defining the experimentally detected intermediate. Additional thermal energy will be then concentrated in the anchoring points of the loops, particularly in the helices 3 and 4, which are the Achilles' heel of the apoflavodoxin core. The distortion of these helices opens the structure and should lead to the final disruption of the three dimensional structure of the protein in longer time scales. Under this general picture, the lack of intermediate when denaturing agent is urea [92] can be easily rationalized, since urea will attack directly the core of the protein [39], eliminating the resistance points that stop the thermal unfolding pathways in a partially disordered conformation.

Under native conditions the thermal intermediate acts as an “in-path” stationary state, since the essential deformations of the intermediate implicitly code the intermediate→native transition, as noted in the high overlap (*Ov* = 0.63; see eq. 3) between the intermediate essential deformation subspace and the intermediate→native transition vector. This finding strongly suggests that the intermediate should be considered as an “activated-high entropy” form of the native state, (see RMSd oscillations in Figure 11), with properties of partially disordered protein, which acts as an attractor of folding routes toward a state that in the absence of an excess of kinetic energy will converge in a down-hill manner to the native form. We can hypothesize that a non-negligible number of partially disordered proteins, which adopt a well-defined three dimensional structure only in the presence of partner, can be considered as generalized examples of three-state folder proteins, which in native conditions populates conformations containing well-structured cores and very mobile regions. The flexibility pattern of such intermediates should favour a down-hill transition to a well-defined three dimensional structure in the presence of interactions stabilizing the disordered region (in these case binding partners).

## Supporting Information

Figure S1 Distribution of different structural descriptors in the meta-trajectory of apoflavodoxin obtained at room temperature. TOP/LEFT: native and total contacts (referred to crystal contacts); TOP/RIGHT: solvent accessible surface (total, hydrophobic and hydrophilic, all in Å^2^); BOTTOM/LEFT: native contacts of structures in the three clusters; BOTTOM/RIGHT: secondary structure content. All reference arrows correspond to crystal values.
(TIF)

Figure S2 Distribution of the Trp solvent accessible (in Å^2^) surface obtained descriptors in the meta-trajectory of apoflavodoxin obtained at room temperature. Reference values correspond to crystal, a highly distorted protein and four fully exposed Trp (see Figure 6 for details).
(TIF)

Figure S3 Distribution of the χ values (the fitting merit function) obtained when MD ensembles of apoflavodoxin at room temperature were used to fit SAXs experimental spectra.
(TIF)

Figure S4 Detail of the loop packing in the crystal lattice of two crystal structures of apoflavodoxin (1FTG, that used as reference here) and 1DX9 (which displays a different crystal symmetry).
(TIF)

Figure S5 Experimental ultraviolet spectra of native, unfolded and intermediate states of apoflavodoxin (see text for details).
(TIF)

Figure S6 Contact plots corresponding to the native and intermediate ensembles. Regions where loss of contacts are especially remarkable are marked.
(TIF)
