# Supplementary figures and images for: Defining the Nature of Thermal Intermediate in 3 State Folding Proteins: Apoflavodoxin, a Study Case
Source: PLoS Comput Biol. 2012 Aug 23;8(8):e1002647. doi: 10.1371/journal.pcbi.1002647 (PMC3426563; doi:10.1371/journal.pcbi.1002647)

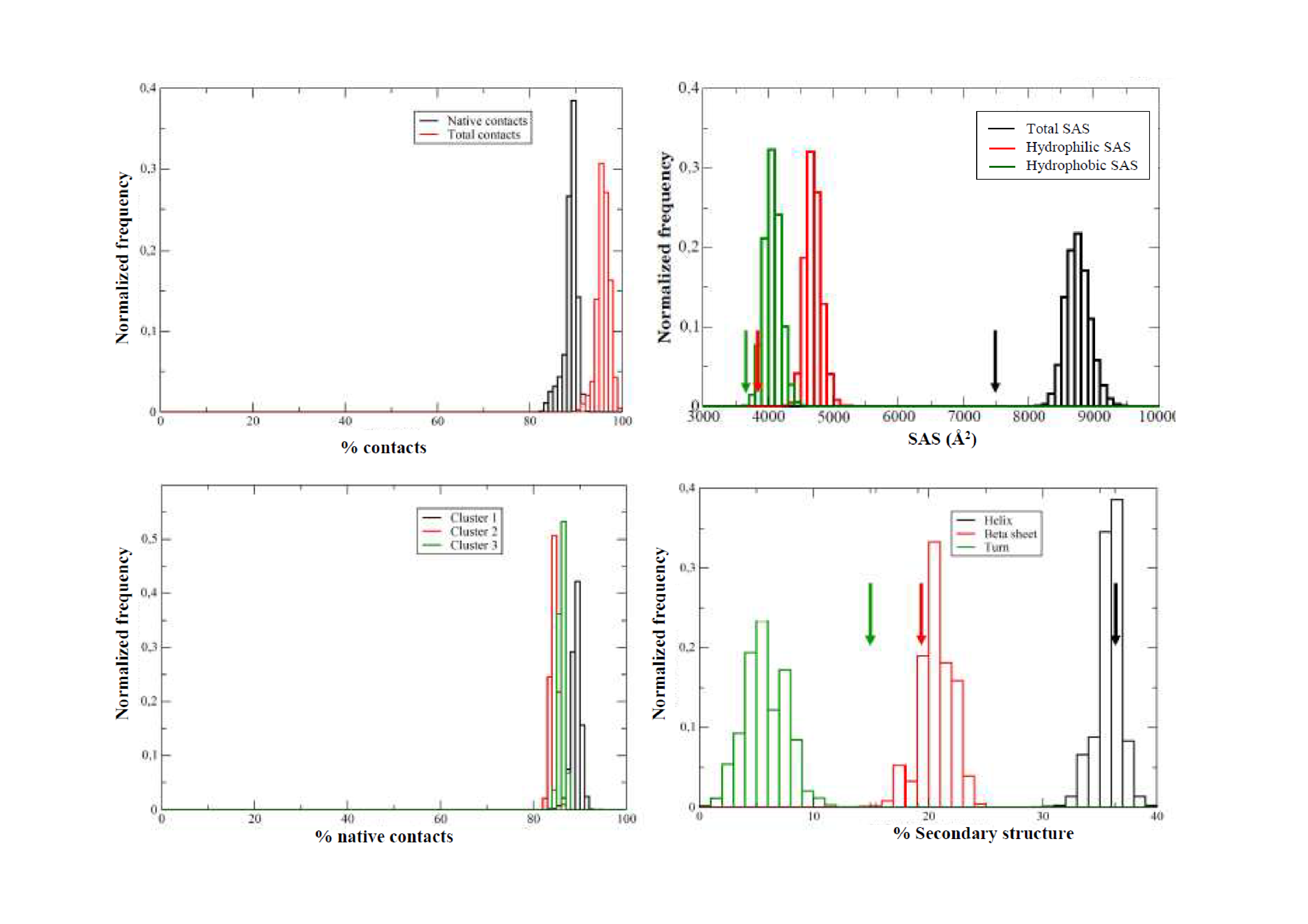

Supplement: Figure S1 — Distribution of different structural descriptors in the meta-trajectory of apoflavodoxin obtained at room temperature. TOP/LEFT: native and total contacts (referred to crystal contacts); TOP/RIGHT: solvent accessible surface (total, hydrophobic and hydrophilic, all in Å2); BOTTOM/LEFT: native contacts of structures in the three clusters; BOTTOM/RIGHT: secondary structure content. All reference arrows correspond to crystal values. (TIF) [file pcbi.1002647.s001.tif]

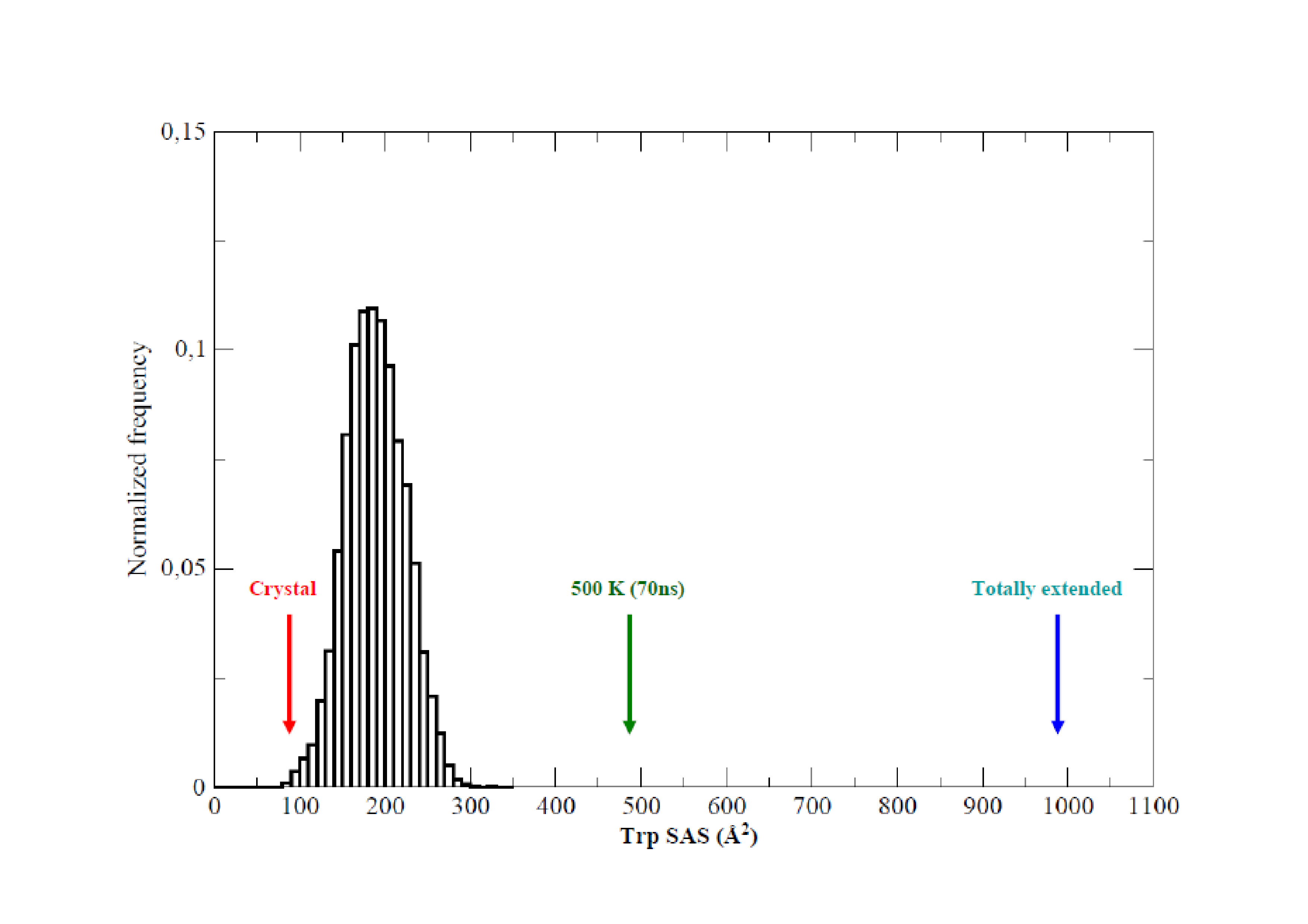

Supplement: Figure S2 — Distribution of the Trp solvent accessible (in Å2) surface obtained descriptors in the meta-trajectory of apoflavodoxin obtained at room temperature. Reference values correspond to crystal, a highly distorted protein and four fully exposed Trp (see Figure 6 for details). (TIF) [file pcbi.1002647.s002.tif]

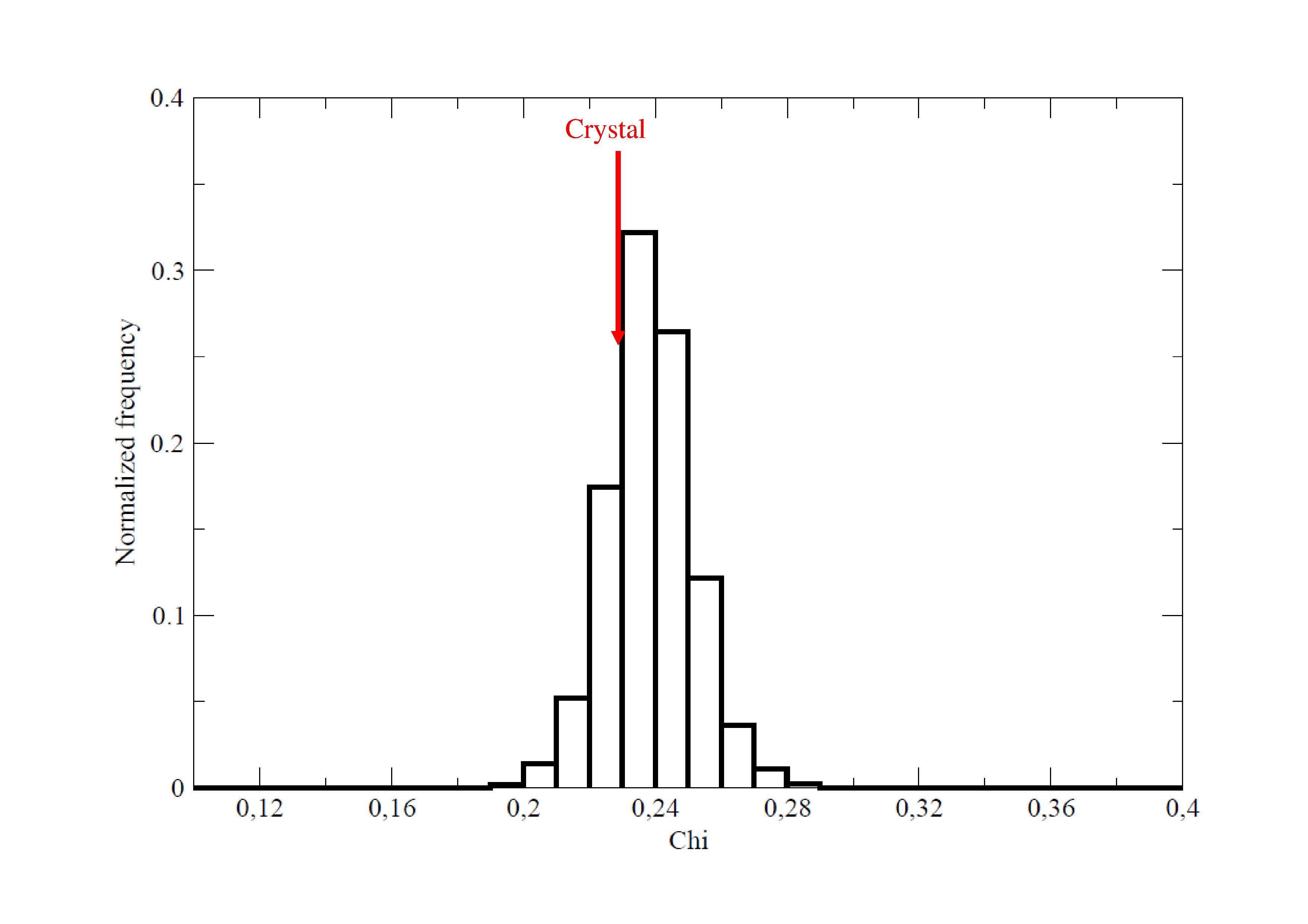

Supplement: Figure S3 — Distribution of the χ values (the fitting merit function) obtained when MD ensembles of apoflavodoxin at room temperature were used to fit SAXs experimental spectra. (TIF) [file pcbi.1002647.s003.tif]

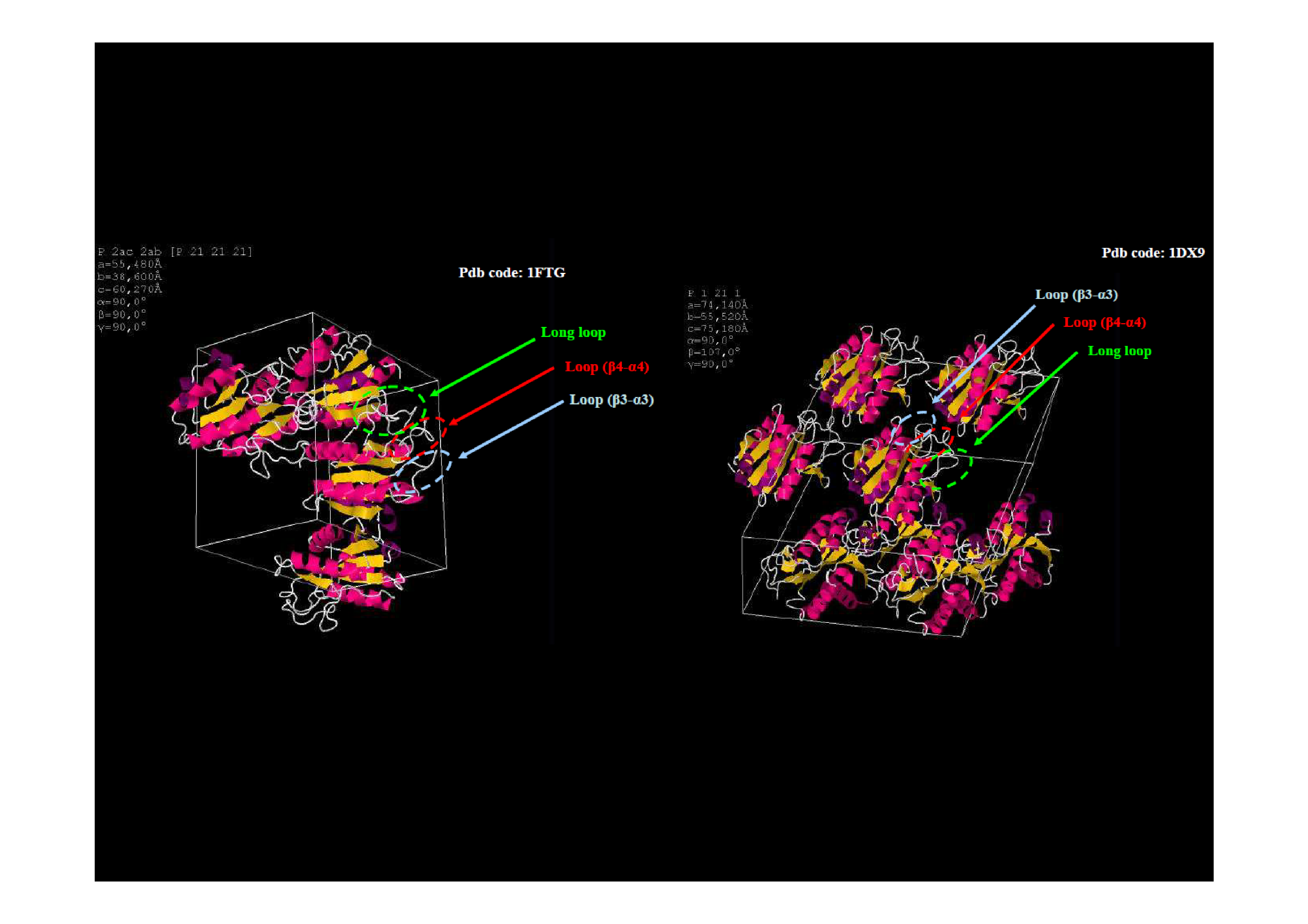

Supplement: Figure S4 — Detail of the loop packing in the crystal lattice of two crystal structures of apoflavodoxin (1FTG, that used as reference here) and 1DX9 (which displays a different crystal symmetry). (TIF) [file pcbi.1002647.s004.tif]

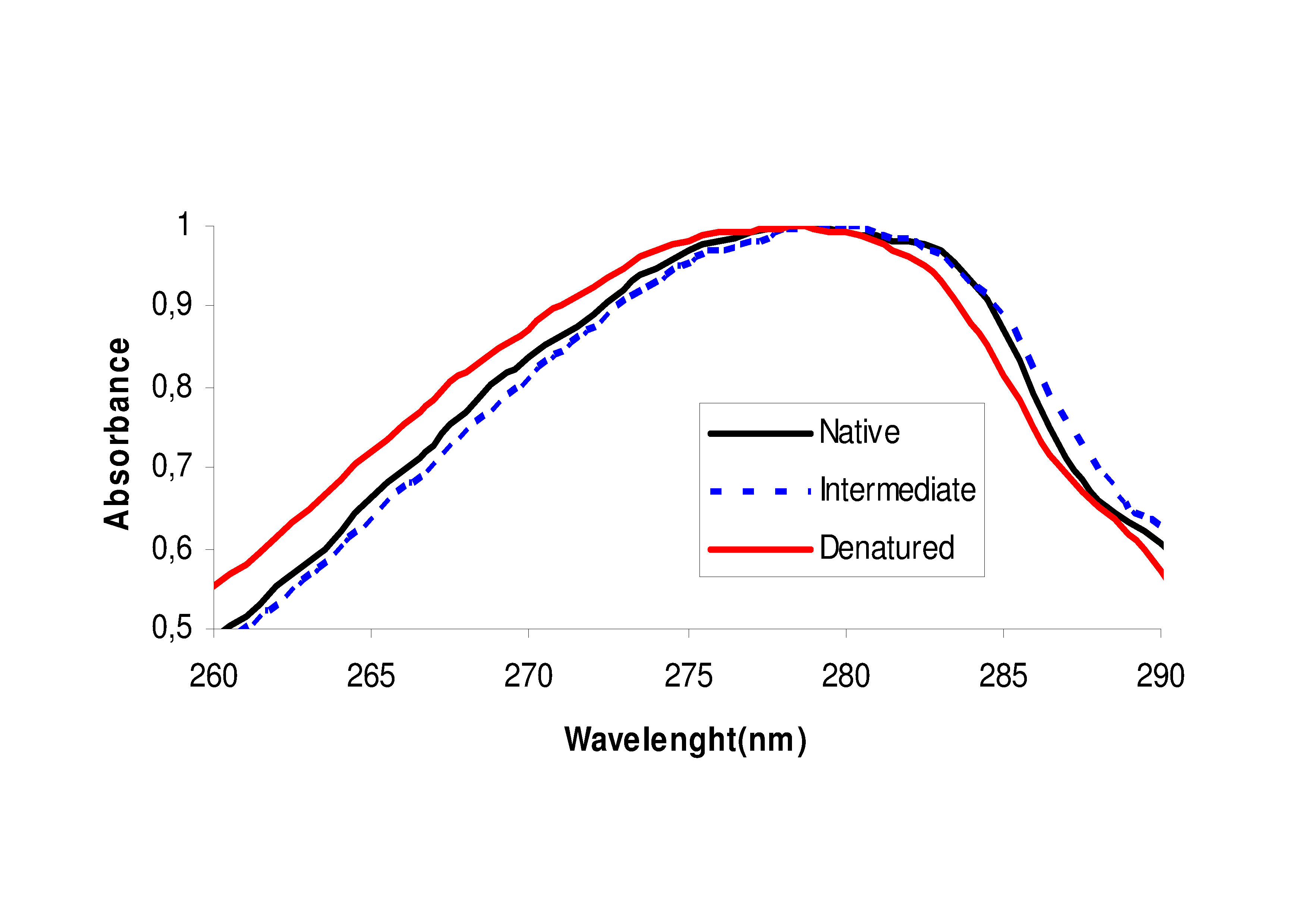

Supplement: Figure S5 — Experimental ultraviolet spectra of native, unfolded and intermediate states of apoflavodoxin (see text for details). (TIF) [file pcbi.1002647.s005.tif]

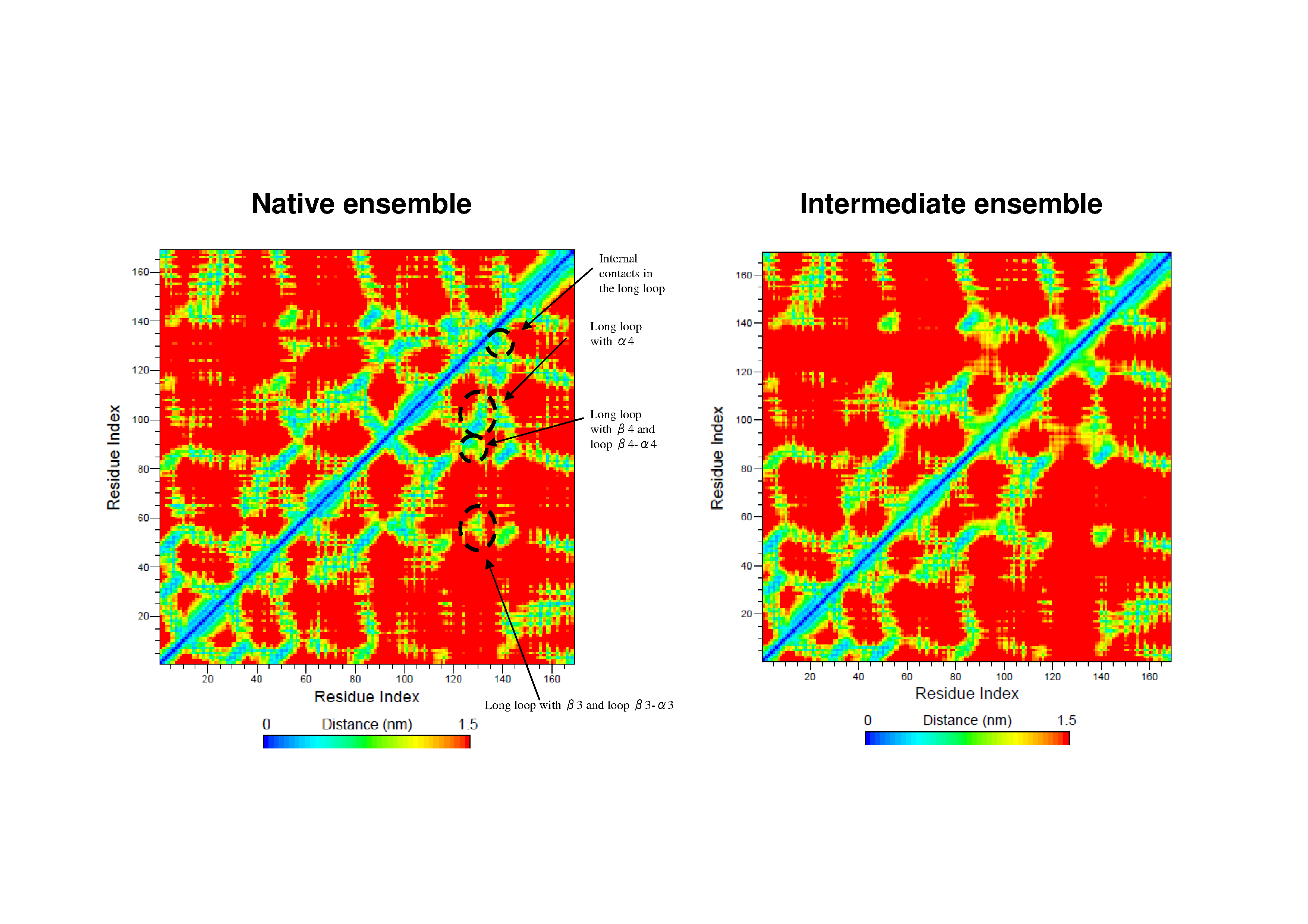

Supplement: Figure S6 — Contact plots corresponding to the native and intermediate ensembles. Regions where loss of contacts are especially remarkable are marked. (TIF) [file pcbi.1002647.s006.tif]
